# Theacrine From *Camellia kucha* and Its Health Beneficial Effects

**DOI:** 10.3389/fnut.2020.596823

**Published:** 2020-12-17

**Authors:** Yue-Yue Sheng, Jing Xiang, Ze-Shi Wang, Jing Jin, Ying-Qi Wang, Qing-Sheng Li, Da Li, Zhou-Tao Fang, Jian-Liang Lu, Jian-Hui Ye, Yue-Rong Liang, Xin-Qiang Zheng

**Affiliations:** ^1^Tea Research Institute, Zhejiang University, Hangzhou, China; ^2^Zhejiang Agricultural Technology Extension Center, Hangzhou, China

**Keywords:** *Camellia sinensis*, antioxidant, anti-inflammation, locomotor, cognition, lipid metabolism, metastasis, hypnosis

## Abstract

Theacrine, i.e., 1,3,7,9-tetramethyluric acid, is one of the major purine alkaloids found in leaf of a wild tea plant species *Camellia kucha* Hung T. Chang. Theacrine has been attracted great attentions academically owing to its diverse health benefits. Present review examines the advances in the research on the health beneficial effects of theacrine, including antioxidant effect, anti-inflammatory effect, locomotor activation and reducing fatigue effects, improving cognitive effect, hypnotic effect, ameliorating lipid metabolism and inhibiting breast cancer cell metastasis effect. The inconsistent results in this research field and further expectations were also discussed.

## Introduction

**Figure 1:**
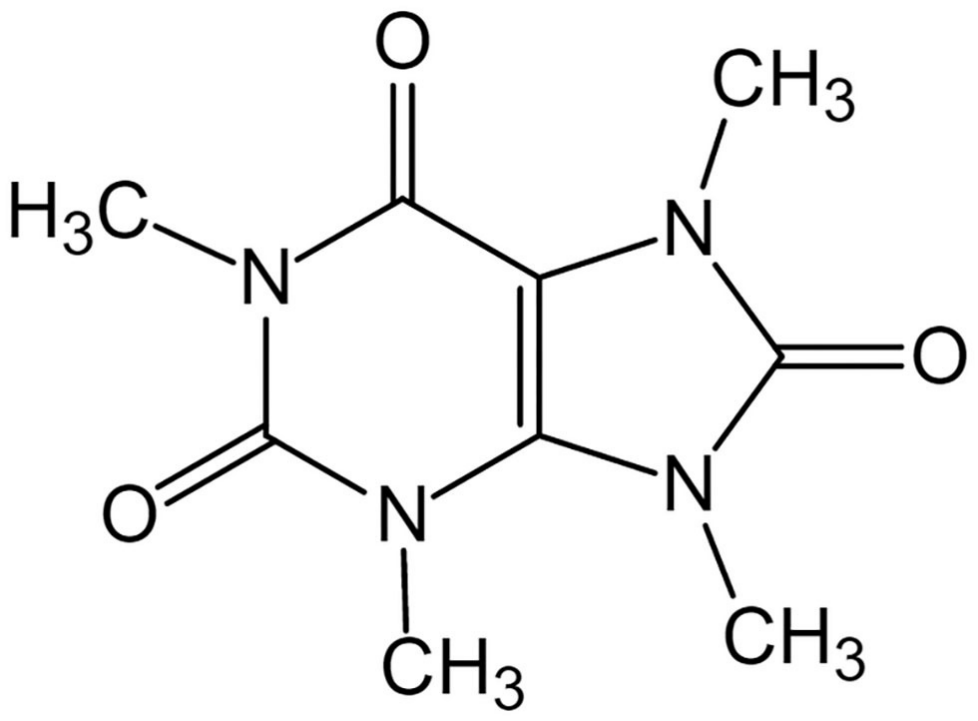
Molecular structure of theacrine.

**Figure 2:**
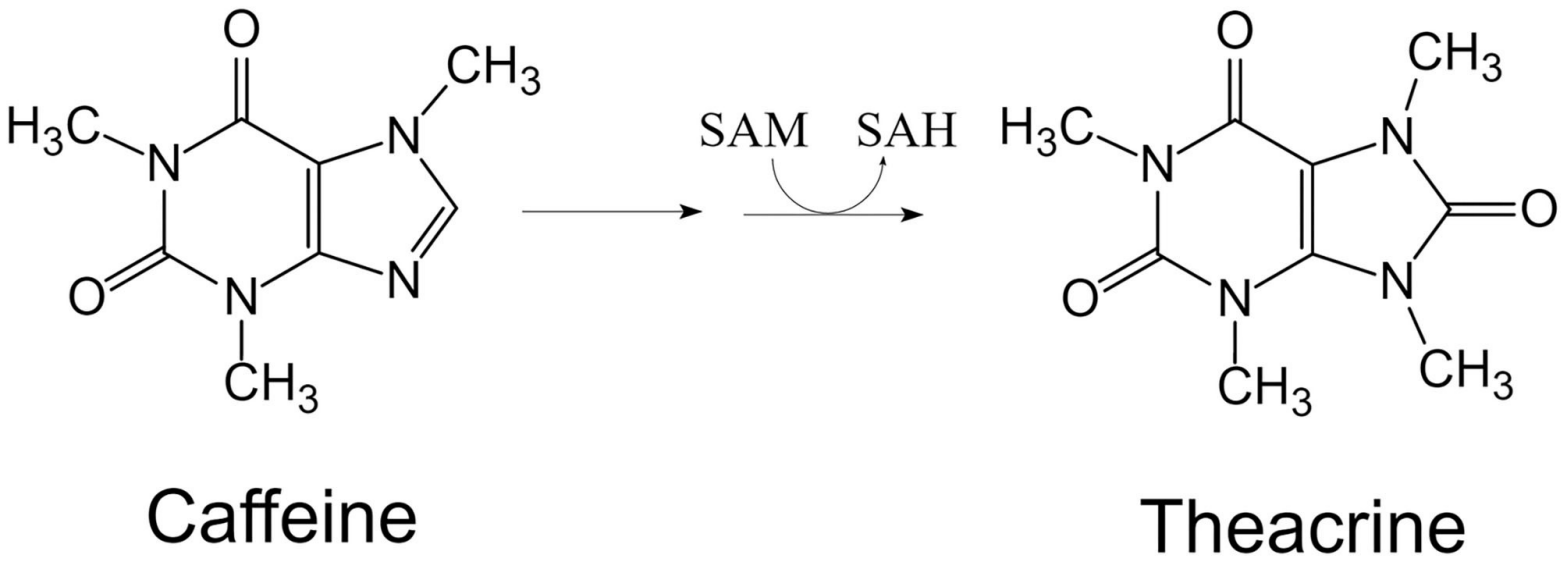
Theacrine is biosynthesized from caffeine catalyzed by N-methyltransferase using SAM as methyl donor. SAM, S-adenosyl-L-methionine; SAH, S-adenosyl-L-homocysteine.

Theacrine is a purine alkaloid found in *Camellia kucha* Hung T. Chang (a wild tea plant species, formerly named as *Camellia assamica* var. *kucha*) (1, 2), and its full name is 1,3,7,9-tetramethyl-1H-purine-2,6,8(3H,7H,9H)-trione, with chemical formula C_9_H_12_N_4_O_3_ and molecular weight 224.22 (Figure 1). It was recently reported that theacrine was detected in *Camellia sinensis var. puanensis* (3)*, Ilex vomitoria* (4), and *Camellia gymnogyna* (5, 6). The content of theacrine in tender shoots with two leaves and a bud of *C. kucha* (Kucha) was 1.3–3.4% based on dry weight (DW). Pure theacrine could be obtained by separating from Kucha leaf through high-speed counter-current chromatography using eluent solvent system composed of hexane/dichloromethane/methanol/water (1/5/4/2, v/v/v/v) (7). Theacrine is a specific purine alkaloid in Kucha and no or little theacrine was detected in the leaf of *Camellia sinensis* which is usually used for processing green tea or black tea. Both theacrine and caffeine are detected in leaf of *C. kucha*. Though it is considered that theacrine is biosynthesized from caffeine catalyzed by N-methyltransferase using S-adenosyl-L-methionine (SAM) as methyl donor (Figure 2), the exact molecular mechanism of theacrine metabolism in Kucha is still unclear (8–10). A theacrine synthase in *C. kucha* (CkTcS) has been identified recently (11). The CkTcS possesses novel N9-methyltransferase activity using 1,3,7-trimethyluric acid but not caffeine as a substrate to biosynthesize theacrine, during which the C8 oxidation of caffeine molecule takes place prior to N9-methylation (8).

Theacrine has been attracted great attentions academically owing to its diverse healthy benefits (1–3), including reducing inflammation (12), providing stimulatory effects without adaptation or habituation (13), antioxidant effect (14), enhancing sustained energy (15), promoting positive mood elevation, and mental clarity (15–17) and *etc*. However, these researches lack systematic summarization and some important mechanisms remain to be further investigated. In this review, we summarized the achievements in the study on health benefits of theacrine and the possible mechanisms behind the observed associations (Table 1), so as to encourage the scientific community to pay more attention to the nutraceutical properties of theacrine.

**Table 1:** Summary of studies on bioactivities of theacrine.

**Bioactivity**	**Assay employed or models**	**Dose or concentration tested**	**Results**	Ref.^*^
Antioxidant	DPPH and FRAP assay	Water soluble Kucha tea extract (TE) 0.2–1.0 mg/mL	The TEAC in scavenging DPPH radicals was 436.78 ± 12.44 mg/mL and IC_50_ was 0.79 ± 0.022 mg/mL; TEAC in scavenging RFAP radicals was 493.67 ± 8.02 mg/mL.	(14)
Antioxidant	ORAC, CAA, chick embryo	50 pM, 50 nM, 50 μM	Compared to AAPH, theacrine significantly increased ORAC and chick embryo vessel density, decreased MDA level.	(18)
Antioxidant	Mouse intervertebral disc degeneration model	Orally 10 mg/kg	Theacrine stimulated the expression of collagen II, SIRT3, FOXO3a, and SOD2.	(19)
Antioxidant	Estrogen-deficient Mouse	20 mg/kg via gavage	Theacrine activated SIRT3 and repress myocardial fibrosis and apoptosis after myocardial infarction in ovariectomized mice, resulting in improvement of the cardiac function of ovariectomized mice with myocardial infarction.	(20)
Antioxidant	Mouse	Orally 10, 20, 30 mg/kg	Theacrine decreased activities of plasma ALT, AST and levels of MDA, but increased activity of SOD and level of ORAC.	(21)
Anti-inflammation	Mouse	Orally 8–32 mg/kg	Theacrine significantly inhibited the ear edema induced by xylene and hind paw edema induced by λ-carrageenan.	(22)
Anti-inflammation	Mouse	Orally 10–30 mg/kg	Theacrine reduced hepatic mRNA levels of inflammatory mediators (IL-6, IL-1 β, TNF α, and IFN γ), decreased the levels of plasma ALT and AST, and reversed the histologic damages.	(21)
Anti-inflammation	FIA-induced SD rats (*n* = 8)	Orally 12.5–25.0 mg/kg	Theacrine attenuated rheumatoid arthritis via the suppression of IL-6 and the activation of TGF-β by the TGF-β/SMAD pathway.	(23)
Locomotor activation and reducing fatigue	Long Evans rats (*n* = 8)	24 or 48 mg/kg, i.p.	Pre-treatment with theacrine significantly attenuated the motor depression induced by the adenosine receptor agonists which is mediated by both the adenosinergic and dopaminergic systems.	(24)
Improving cognitive performance	Clinic test on Human (*n* = 12)	Orally 65–300 mg theacrine and 25–650 mg caffeine daily	Administration of the theacrine and caffeine improved mood, energy, focus, concentration, cognitive function, or sexual desire, reduced anxiety or fatigue.	(25, 26)
Improving cognitive performance	Clinic test on Human (*n* = 19)	Orally PWS containing 10–15 mg theacrine for 1 week	Theacrine significantly improved Stroop word testing and Stroop color testing results.	(27)
Improving cognitive performance	6-OHDA-treated rats and MPTP-treated mice and zebrafish	Theacrine 10–20 mg/kg, i.p.	Theacrine relieved PD by retrieving the apoptosis of dopaminergic neurons and activating SIRT3.	(28)
Improving cognitive performance	Male kunming mice/*Morris* water maze test	Orally 5–15 mg/kg,	Theacrine significantly reversed learning and memory impairment caused by central fatigue by normalizing glucose metabolism, restoring 5-HT level and inhibiting phosphodiesterase in the restraint-stressed mice brain.	(17)
Improving cognitive performance	Male and female soccer players (*n* = 24)	Orally 275 mg placebo (PL), 275 mg Teacrine (TCr), 275 mg caffeine (Caf), or a combination of 125 mg TCr and 150 mg Caff (TCr + Caf) 30 min prior to exercise.	Compared to PL, 275 mg of Caf or a combination of Caf + TCr produced modest cognitive benefits, particularly following the first half of the simulated soccer match.	(16)
Hypnotic and antidepressant effects	Mice (*n* = 20)	Orally 10 and 30 mg/kg	Theacrine prolonged remarkably pentobarbital-induced sleeping time at both doses (*p* < 0.01).	(29)
Hypnotic and antidepressant effects	Male NIH mice (*n* = 10)	3, 10, 30 mg/kg, i.g.	Theacrine showed antidepressant effects on mice, including shortening the immobility time in the tail suspension test (*P* < 0.05), enhancing the rotatory locomotor activities during forced swimming test (*P* < 0.05), increasing the mortality induced by yohimbine and the head-twitching number induced by 5-HTP (*p* < 0.01).	(30)
Hypnotic and antidepressant effects	Male ICR mice and male Sprague Dawley rats (*n* = 5)	3, 10, 30 mg/kg, i.g.	Theacrine significantly enhanced pentobarbital-induced sleep in mice by shortening wake time and increasing NREMS time, but it had no effect on REMS. Theacrine significantly reversed the decrease in sleeping time in mice pretreated with the adenosine A1 receptor antagonist DPCPX.	(31)
Ameliorating lipid metabolism	*In vitro*: 3T3-L cells; *in vivo*: kunming mice (n-8)	*In vitro*: 2, 0.5, 0.125, 0.03125 m mol/L; *in vivo*: orally 0, 20, 40 mg/kg	*In vitro*, theacrine up-regulated expression of genes encoding SIRT3, LCAD, FABP4 and LPL genes. *In vivo*, theacrine (10 mg/kg) up-regulated SIRT3 gene expression in liver, but 40 mg/kg of theacrine showed inhibition on SIRT3.	(32)
Ameliorating lipid metabolism	3T3-L cells	50–200 μg/Ml *C. kucha* extract	Kucha extract significantly suppressed lipid droplet and TG accumulation and inhibited the expression of genes FAS, FAT, SCD-1, LPL, SREBP-1c and ACC in 3T3-L1 cells in dose dependent manner.	(33)
Ameliorating lipid metabolism	*In vivo*: C57BIM 6J mice (n-8); *in vitro*: HepG cells	Theacrine 10, 20 mg/kg for *in vivo* and 2 m mol/L for *in vitro*.	Theacrine significantly decreased hepatic TG content (*p* < 0.01) and ameliorated hepatic steatosis in mice.	(34)
Ameliorating lipid metabolism, anti-inflammation	C57BL/6 mice	Theacrine 10, 20 mg/kg	Theacrine inhibited hepatic steatosis and liver inflammation, improved energy expenditure and ameliorated acylcarnitine metabolism disorder in HFD-fed mice through SIRT3/LCAD signaling pathway.	(35)
Inhibiting cancer cell metastasis	MDA-MB-231 cells	Theacrine 10–100 μM	Theacrine suppressed TGF-β-induced progression and metastasis of TNBC through inhibiting EMT.	(36)
Anti-virus	MDCK cells	50 or 100 μM	Theacrine at 50 μM significantly inhibited the expression of influenza virus A NS1 protein at 12 hpi; theacrine at 100 μM significantly reduced the yield of viral progenies.	(37)
No effect	Men (*n* = 10) and women (*n*-10)	Orally TheaTrim^TM^ containing 150 mg theacrine	Theacrine did not result in a statistically significant improvement in cognitive performance, but might favorably impact multiple subjective feelings related to energy and mood.	(38)
No effect	Resistance-trained men (*n* = 12)	Theacrine 300 mg, caffeine 300 mg, or theacrine 150 mg + caffeine 150 mg (combination)	Neither theacrine, caffeine or their combination improved muscular strength, power, or endurance performance in resistance-trained men.	(39)

* *Ref., Reference*.

## Toxicological Evaluation of Theacrine

Acute toxicity test (14 d) on mice using extracts of Kucha showed that the semi-lethal dose (LD_50_) of Kucha extracts and theacrine was 5,600 mg/kg bw/day (12) and 810.6 mg/kg bw/day (22), respectively. Four-week oral administration of pure theacrine by rats at dose up to 150 mg/kg bw/day, showed that the appearance and behavior, body weights, organ coefficient, hematological and biochemical parameters of the tested rats showed as normal as control group. Pathological examination showed no changes induced by the drug toxicity, and no delayed toxic reaction was observed after stopped giving drug (40). A 90-day oral toxicological evaluation showed that the no observed adverse effect level (NOAEL) of theacrine was 180 mg/kg bw/day, as at this dose there were no toxicologically relevant treatment-related findings in male or female animals (41).

Oral administration test on sixty healthy men and women using TeaCrine®, a nature-identical, chemically equivalent bioactive version of theacrine, confirmed that daily supplementation theacrine up to 300 mg/kg bw/day for more than 8 weeks was clinically safe, without habituating neuro-energetic effects (13). Tests on one-hundred twenty-five men and women (mean age 23.0 years, height 169.7 cm, body mass 72.1 kg; *n* = 25 persons/group) showed that oral consumption of a mixture containing methylliberine (100–150 mg) and theacrine (25–50 mg) showed no negative effect on the health over 4 weeks of continuous oral administration (42).

## Health Beneficial Effects of Theacrine

### Antioxidant Effects

The leaf of *C. kucha* containing 2.86% (W/W) theacrine and 2.13% (W/W) caffeine had stronger antioxidant activity than *Camellia sinensis* (Longjing tea) containing 4.64% (W/W) caffeine but no theacrine (14). The IC_50_ value (inhibitory concentration in mg/mL of tea necessary to reduce the absorbance by 50%) of *C. kucha* in scavenging DPPH radicals was 0.79 ± 0.022 mg/mL, being lower than that of *C. sinensis* (0.91 ± 0.015 mg/mL). The TEAC value (Trolox equivalent antioxidant capacity, TEAC (mg/g) = IC_50(trolox)_/IC_50(sample)_ × 1,000) of *C. kucha* in scavenging DPPH radicals (436.78 ± 12.44 mg/g) was higher than that of *C. sinensis* (376.12 ± 5.81 mg/g) (14). The TEAC value of *C. kucha* in FRAP assay (493.67 ± 8.02 mg/g) was also higher than that of *C. sinensis* (438.05 ± 9.54 mg/g) (14). These suggest that *C. kucha* containing theacrine had stronger DPPH and FRAP radical scavenging activities than *C. sinensis* without theacrine. Purine alkaloids including caffeine, theophylline, theobromine and theacrine, which are detected in *C. kucha*, showed comparatively lower antioxidant activities in oxygen radical absorbance capacity (ORAC) and cellular antioxidant activity assay (CAA) than Trolox. The ORAC values of theabromine, theophylline, caffeine and theacrine were ~65, ~37, ~35, and ~25% of that of Trolox (18). Though theacrine showed lower activity in ORAC and CAA than theophylline and theobromine, it could significantly improve vessel density on chorioallantoic membrane and myocardial apoptosis in chick embryo model (18). These properties are related to its upregulating expression of sirtuin which is a group of highly conserved NAD-dependent histone deacetylases in mammals and has regulatory effects on cell survival, proliferation, metabolism, death and aging, as well as longevity of organs. Theacrine upregualted the expression of sirtuin family member SIRT3, which in turn activated the SIRT3/FOXO3/SOD2 signaling pathway, resulting in improvement of the intervertebral disc degeneration (19) and the cardiac function of ovariectomized mice with myocardial infarction (20) by alleviating oxidant stress. Furthermore, the antioxidant capacity of theacrine was also due to its strengthening the antioxidant system *in vivo*, such as elevating activities and gene expressions of superoxide dismutase (SOD), catalase, and glutathione peroxidase (GSH-Px), as well as reducing activity of xanthine oxidase, resulting in significant decrease in the content of malondialdehyde (MDA) and increase in ORAC level in plasma and liver of oxidant-stressed mice (21).

### Anti-inflammatory Effects

Oral administration of theacrine by mice (8–32 mg/kg bw/day) significantly inhibited the ear edema induced by xylene and hind paw edema induced by λ-carrageenan in a dose dependent manner (22). Oral administration of theacrine showed potent anti-inflammatory activities. Theacrine inhibited the xylene-induced ear edema, in which the inhibition percentages were 10.1, 12.9, and 45.2% at doses 8, 16, and 32 mg/kg, respectively, compared with the control. Maximal inhibitory rates of anti-edema effect of theacrine at doses 8, 16 and 32 mg/kg were observed at the 2 h, with inhibitory rates being 14.1, 16.6, and 20.1% (*P* < 0.05), respectively, compared with 18.8% of indomethacin (22). Theacrine showed inflammatory cell infiltration and focal necrosis of hepatocytes. Oral administration of theacrine (10, 20, 30 mg/kg DW/day) for 7 consecutive days was found to reduce hepatic mRNA levels of inflammatory mediators (IL-6, IL-1β, TNF α, and IFN γ), decreased the levels of plasma alanine aminotransferase (ALT) and aspartate aminotransferase (AST), and reversed the histologic damages in liver of restraint stressed mice (43). Oral administration of theacrine (12.5–25.0 mg/kg bw/day) showed therapeutic effect on arthritis in SD rat (43). An aqueous formula consisting of bioactive form of theacrine, liberine and methylliberine was used as dietary supplement for reducing inflammation, pain, depression, and alleviating deleterious effects of hyperglycemia and sleep deprivation (43).

Theacrine shows anti-inflammation effect by interfering with the actions of inflammatory mediators such as histamine, serotonin, and bradykinin (22). The inflammatory mediators histamine, serotonin, and bradykinin induce edema by promoting vasodilatation and increasing vascular permeability (44). λ-Carrageenan-induced edema is a biphasic event, in which the early phase (90–180 min) of the inflammation is due to the release of histamine, serotonin and similar substances, and the later phase (270–360 min) is associated with the activation of kinin-like substances (45). Theacrine functions on the first phase of inflammation by interacting with histamine and serotonins and other similar mediators. Furthermore, theacrine has a membrane stabilizing effect, results in reduction of capillary permeability (22). The anti-inflammation mechanism of theacrine is also involved in its enhancement of transforming growth factor-β (TGF-β) mediated shifts *via* TGF-β/SMAD pathway (23). Biological signals for TGF-β are transduced through transmembrane serine/threonine kinase receptors to a family of intracellular mediators known as SMADs. Theacrine upregulated the expression of RNA and protein of SMAD3, p-ERK and p-p38, but downregulated the expression of nuclear factor-kB (NF-kB) and interleukin-6 (IL-6) in Freund's incomplete adjuvant (FIA)-induced SD rats (23).

### Locomotor Activation and Reducing Fatigue

There were many studies showing that theacrine activated locomotor and reduced fatigue. In comparison to placebo under double-blind conditions, oral administration of TeaCrine® at a dose equivalent 200 mg theacrine resulted in more energy, less fatigue, better concentration and mood (15). Dietary supplement with 5–850 mg theacrine would improve mood, energy and focus, accompanying with reduction of anxiety or fatigue (25). A formula containing theacrine and caffeine with weight ratio 2:1 to 4:1 induced an increase in energy of at least 8%, accompanying with a decrease in fatigue at least 6% (26). A pre-workout supplement of 10 mg theacrine improved selective attention (27). 27–38% improvements in time-to-exhaustion were observed in participants with supplement of 275 mg theacrine, leading athletes to sustain greater focus under fatigue for long periods (16). Sense of energy was significantly increased from baseline to 2 h post-ingestion in treatments of caffeine and combination of theacrine + caffeine; meanwhile, oral administration of 125 mg theacrine produced a significant rise in energy in 3 h post-ingestion, with little impact on heart rate (HR) and blood pressure (BP) (17). The mechanism of theacrine activating locomotor and reducing fatigue is likely involved in its action as an adenosine receptor antagonist. There was study showing that pre-treatment with theacrine (24 or 48 mg/kg, i.p.) significantly attenuated the motor depression induced by the adenosine receptor A_1_ agonist (N^6^-cyclopentyladenosine, CPA; 0.1 mg/kg, i.p.) and receptor A_2A_ agonist (2-p-(2-carboxyethyl)phenethylamino-5′-N-ethylcarboxamidoadenosine; 0.2 mg/kg, i.p.), but this effect was reduced by both antagonists D1R SCH23390 (0.1 or 0.05 mg/kg, i.p.) and D2R eticlopride (0.1 mg/kg, i.p.), indicating that theacrine is likely acting as an adenosine receptor antagonist (24).

### Improving Cognitive Performance

Test on mice showed that oral administration of theacrine (5, 10, 15 mg/kg) significantly reversed learning and memory impairment caused by central fatigue (28). Combination of theacrine (125 mg) and caffeine (150 mg) moderately improved cognitive performance as assessed by trail making test (TMT) (17) and showed modest cognitive benefits during complex decision making, potentially due to overlapping peak concentrations or enhanced bioavailability (26, 27). The improved cognitive accuracy at end-of-game in all conditions may indicate a training effect in highly skilled players for allocation of resources (16).

The cognitive improving effect of theacrine was associated with its regulating brain glucose metabolism, inhibiting phosphodiesterases and restoring levels of fatigue-related neurotransmitters in the brains, such as 5-hydroxytryptamine (5-HTP) and dopamine as well as their metabolites (28).

Parkinson's disease (PD) is a long-term degenerative disorder of the central nervous system that mainly affects the motor system, affecting about 1% of adults older than 60 years (28). Oxidative damage of dopaminergic neurons is the fundamental causes of PD. Tests using multiple animal models of PD (6-OHDA-treated rats and in 1-methyl-4-phenyl-1,2,3,6-tetrahydropyridine (MPTP)-treated mice and zebrafish) and 1-methyl-4-phenylpyridinium (MPP^+^)-treated SH-SY5Y cells showed that theacrine had the potency to relieve PD by retrieving the apoptosis of dopaminergic neurons and activating SIRT3 which deacetylating SOD2 and restoring mitochondrial functions (46).

### Hypnotic and Antidepressant Effects

Theacrine is structurally similar to caffeine (Figure 2), but in fact its physiological effects are quite different from the latter. Theacrine is shown to be stimulatory when used in higher doses, however, it can actually have a sedative effect when used in low doses such as the amount consumed through tea leaves of *C. kucha*. Oral administration of theacrine (10 and 30 mg/kg) could significantly prolong the sleeping time induced by pentobarbital (*p* < 0.01), while caffeine and theobromine exhibited an inverted effect on mice. This suggests that theacrine possesses potent sedative and hypnotic properties and its central nervous system effects is different from those of caffeine and theobromine (29). Furthermore, caffeine has been demonstrated to show signs of tolerance build-up in as little as 4 days, while theacrine shows no such adaptation (13).

Intragastric administration of theacrine (3, 10, 30 mg/kg BW/day) showed antidepressant effects on mice, including shortening the immobility time in the tail suspension test (*P* < 0.05), enhancing the rotatory locomotor activities during forced swimming test (*P* < 0.05), increasing the mortality induced by yohimbine and the head-twitching number induced by 5-HTP (*p* < 0.01). Theacrine could also markedly improve such symptoms as hypothermia (*P* < 0.01), akinesia (*P* < 0.05), and eye ptosis of mice (*P* < 0.01) induced by reserpine, which may be contributed to its influence on monoamine neurotransmitter (30). Sleep parameter analysis by electroencephalogram (EEG) and electro-magnetic gun (EMG) showed that theacrine (3.0 mg/kg, i.g.) significantly enhanced pentobarbital-induced sleep in mice by shortening wake time and increasing non-rapid-eye-movement sleep (NREMS) time, but it had no effect on rapid-eye-movement sleep (REMS). Meanwhile, theacrine markedly attenuated caffeine (a non-selective antagonist of adenosine receptor)-induced insomnia (31). Theacrine worked as a non-selective adenosine receptor agonist to induce a hypnotic effect through the adenosine system. There was study showing that theacrine significantly reversed the decrease in sleeping time in mice pretreated with the adenosine A_1_ receptor antagonist 8-cyclopentyl-1,3-dipropylxanthine (DPCPX) and the A_2A_ receptor antagonist 7-(2-phenylethyl)-5-amino-2-(2-furyl)-pyrazolo-(4,3-e)-1,2,4-triazolo[1,5-c]pyrimidine (SCH 58261), during which theacrine markedly increased the adenosine content but inhibited adenosine deaminase activities in the hippocampus of rats (31). These suggested that theacrine mediates the adenosine system to augment pentobarbital-induced sleep.

### Ameliorating Lipid Metabolism

An *in vitro* test showed that theacrine up-regulated SIRT3, LCAD, FABP4 and LPL gene expression (32). *In vivo* test showed that theacrine (10 mg/kg) up-regulated SIRT3 gene expression in liver, but 40 mg/kg of theacrine showed inhibition on SIRT3. Low-dose theacrine (10 and 20 mg/kg) up-regulated hepatic LCAD, FABP4, HTGL, and ATP5c mRNA expression, while high-dose theacrine (40 mg/kg) down-regulated their levels. It shows that theacrine has an effect on fatty acid metabolism through regulating fatty acid metabolism related genes, and the mechanism may be related with its up-regulation of SIRT3 expression (32). Extract (50–200 μg/mL) of *C. kucha* with 1.44% (W/W/) theacrine showed significant suppressive effect on lipid droplet accumulation and triglyceride (TG) level in 3T3-L1 cells in a dose dependent manner by suppressing the expression of genes involving in lipid metabolism, including FAS, FAT, SCD-1, LPL, SREBP-1c, and ACC (33). Test on C57B/6J mice showed that theacrine significantly decreased hepatic TG content (*p* < 0.01) and ameliorated hepatic steatosis in mice (34). Acylcarnitine metabolism disorder contributes significantly to the pathogenesis of non-alcoholic fatty liver disease (NAFLD). Theacrine ameliorated acylcarnitine metabolism disorder in high-fat diet (HFD)-fed mice, resulting in suppression of hepatic steatosis and liver inflammation and improvement of energy expenditure through the SIRT3/LCAD signaling pathway (35).

The underlying mechanism that theacrine ameliorates high fat diet induced hepatic steatosis in mice involves in SIRT3/AMP-activated protein kinase (AMPK)/acetyl-CoA carboxylase (ACC) pathway, during which the activation of ACC by theacrine depends on the phosphorylation of AMPK, but the activation of SIRT3 by theacrine is independent of the phosphorylation of AMPK (34). Theacrine promotes acylcarnitine metabolism in non-alcoholic fatty liver disease (NAFLD) through the SIRT3/LCAD signaling pathway, in which the target of theacrine's activities on NAFLD is identified as SIRT3 (35). Theacrine up-regulated the expression of SIRT3 as well as the phosphorylation of AMPK and ACC (28). Theacrine activated the mitochondrial deacetylase SIRT3 and consequently, the increased activity of long-chain acyl coenzyme A (CoA) dehydrogenase (LCAD) through deacetylation (32, 35). Peroxisome proliferator-activated receptor γ (PPAR γ) and CCAAT/enhancer-binding protein α (C/EBP α) are considered to be the key regulators of adipogenesis (47, 48). Theacrine suppressed the lipid droplet accumulation in 3T3-L1 adipocytes by downregulation of the expression of major transcription factors of adipogenesis pathway including PPAR γ and C/EBP α (33). Furthermore, theacrine also decreased the mRNA and protein levels of fatty acid synthase, fatty acid translocase, steroylcoenzyme A desaturase-1, lipoprotein lipase and acetyl-CoA carboxylase-1 (33).

### Inhibiting Breast Cancer Cell Metastasis

Theacrine also inhibited transforming growth factor-β (TGF-β) induced cell adhesion, migration, and invasion, showing antimetastatic potential implications for disease management in breast cancer (36). Epithelial-to-mesenchymal transition (EMT), whereby epithelial cells transform into mesenchymal cells, is shown to be related to cancer and cancer cell metastasis. The EMT process is coordinated by the loss of expression of epithelial marker such as Occludin and E-cadherin, accompanying with gain of mesenchymal markers such as Vimentin, Fibronectin and N-cadherin, which promote tumor progression, cell invasion, and metastasis (49). The EMT markers expression is associated with poor prognosis of breast cancer patients (50, 51). TGF-β-induced EMT has been recognized as an emerging mechanism underlying its metastasis-promoting function (52). The regulators of EMT have become attractive targets for developing anti-metastasis therapies. Theacrine reversed EMT, resulting in downregulation of mesenchymal markers including Fibronectin, Vimentin, N-cadherin, Twist, and Snail and upregulation of epithelial markers such as Occludin and E-cadherin in human breast cancer MDA-MB-231 cells. Also, theacrine attenuated TGF-β-induced EMT, cell adhesion, migration, and invasion in the MDA-MB-231 cells. These suggest that theacrine shows suppressive effects on TGF-β-induced progression and breast cancer cell metastasis by reversing the EMT process (36).

Furthermore, there was study revealing that theacrine showed anti-virus activity. Theacrine at 50 μM dosage significantly inhibited the expression of NS1 protein in human influenza virus A/Puerto Rico/8/34 at 12 hpi, and 100 μM theacrine significantly suppressed the virus progeny production (37).

## Conclusions and Future Expectations

**Figure 3:**
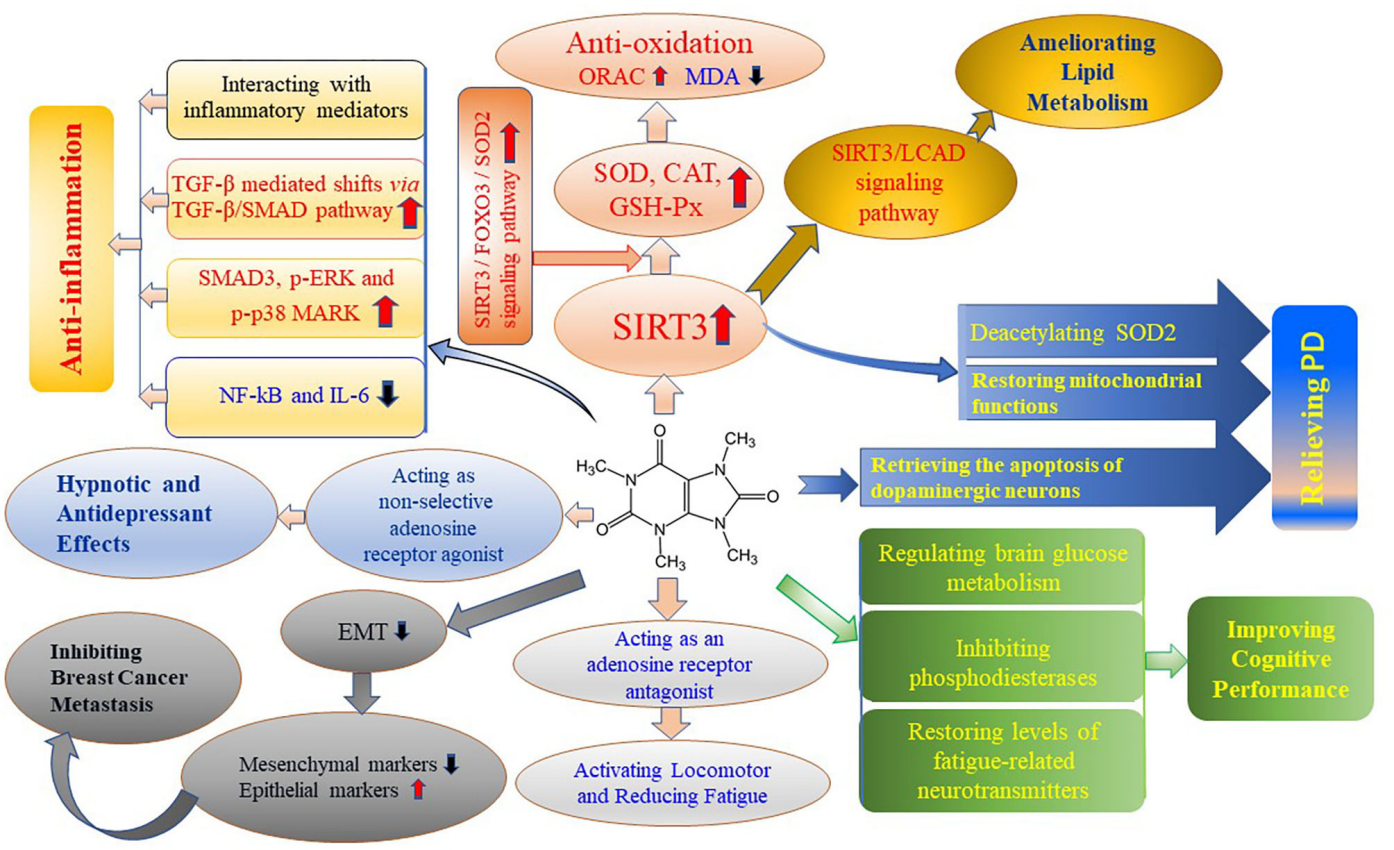
Bioactivities of theacrine. The end of each direction shows one bioactivity of theacrine, in which the intermediates show the actions of theacrine or signaling pathways. CAT, Catalase; EMT, Epithelial-to-mesenchymal transition; FOXO3, Forkhead Box O3, a protein coding gene, including DNA-binding transcription factor activity and protein kinase binding; GSH-Px, Glutathione peroxidase; IL-6, interleukin-6; LCAD, Lightweight chemical agent detector; NF-kB, Nuclear factor-kb; ORAC, oxygen radical absorbance capacity; PD, Parkinson's disease; p-ERK, phosphorylated extracellular regulated protein kinases; p-p38 MARK, phosphorylated mitogen-activated protein kinases; MDA, malondialdehyde; SIRT3, Sirtuin-3, a major mitochondria NAD^+^-dependent deacetylase; SMAD, the proteins are required for the transmission of the TGF-β signal to the nucleus; SOD, Superoxide dismutase; TGF-β, Transforming growth factor-β. The red up arrow means increase in the indicators and the blue down arrow means decrease in the indicators.

Theacrine is a purine alkaloid found in *Camellia kucha* and it shows diverse health benefits. Theacrine upregulates the expression of SIRT3 and activates the SIRT3/FOXO3/SOD2 signaling pathway, resulting in antioxidant efficiency. Theacrine upregulates the expression of RNA and protein of SMAD3, p-ERK and p-p38 and downregulates the expression of NF-kB and IL-6, showing anti-inflammatory effects. Theacrine acts as an adenosine receptor antagonist to play a role in the locomotor activation and fatigue reduction. Theacrine regulates brain glucose metabolism, inhibits phosphodiesterases and restores the levels of 5-HTP and dopamine to improve cognitive capacity. Theacrine possesses potent sedative and hypnotic properties and its central nervous system effects through mediating the adenosine system. Theacrine ameliorates lipid metabolism by activating SIRT3/AMPK/ACC pathway and downregulating the mRNA and protein levels of fatty acid synthase, fatty acid translocase, steroylcoenzyme A desaturase-1, lipoprotein lipase and acetyl-CoA carboxylase-1. Theacrine shows suppressive effects on TGF-β-induced progression and breast cancer cell metastasis by reversing EMT, which in turn leads to the downregulation of mesenchymal markers and upregulation of epithelial markers, and also attenuating TGF-β-induced EMT, cell adhesion, migration, and invasion. The bioactivities of theacrine are shown in Figure 3.

However, there is still a long way to go to the clinical application of theacrine. First, the mechanism underlying the physiological activities of theacrine remains to be further explored. Theacrine has similar chemical structure as caffeine, but how their physiological activities are differentiated greatly? The mechanism why theacrine was stimulatory at higher doses, but it could actually have a sedative effect at low doses remains to be clarified (30). Further studies need to elucidate the mechanisms by which theacrine downregulated the EMT markers (36). Second, drug–drug interaction between theacrine and other compounds needs to be further investigated. It was found that co-administration of theacrine and caffeine resulted in a clinically significant pharmacokinetic interaction, viz., caffeine co-administration increased maximum plasma concentration and the area under the curve of theacrine without altering theacrine half-life in comparison with the corresponding pharmacokinetic parameters when theacrine is administered alone (53). There was pharmacokinetic interaction between methylxanthines and theacrine in humans when theacrine (50 mg), methylliberine (100 mg) and caffeine (150 mg) were orally administered (54). More work needs to probe how the enhanced oral bioavailability was happened. Third, some inconsistent results remain to be reconfirmed. It was reported that acute intake of the theacrine-containing dietary supplement did not improve cognitive performance statistically though it favorably impacted multiple subjective feelings related to energy and mood (38). There was study showing that neither theacrine (300 mg), caffeine (300 mg) and nor combination of both theacrine (150 mg) and caffeine (150 mg) improved muscular strength, power, or endurance performance in resistance-trained men when consumed 90 min pre-exercise (39). Caffeine treatment led to a marked increase in the ambulatory activity accompanied with decreasing of the immobility time in forced swimming test at both 10 and 30 mg/kg. Under the same conditions, neither theacrine nor theobromine showed obvious excited efficacy (29, 55). The clarification of these issues would be greatly helpful for developing clinical applications of theacrine.

## Author Contributions

Y-RL, Y-YS, and X-QZ conceived the project and references collection. Y-YS introduction. JX toxicological evaluation of theacrine. Z-SW and JJ anti-oxidant effects. Y-QW anti-inflammatory effects. Q-SL and Z-SW locomotor activation and reducing fatigue. Z-TF and DL improving cognitive performance. J-LL and J-HY ameliorating lipid metabolism. Y-RL inhibiting breast cancer cell metastasis and abstract. X-QZ conclusions and future expectations. All authors contributed to the article and approved the submitted version.

## Conflict of Interest

The authors declare that the research was conducted in the absence of any commercial or financial relationships that could be construed as a potential conflict of interest.

## Abbreviations

5-HT: 5-Hydroxytryptamine
5-HTTP: 5-Hydroxytryptophan
6-OHDA: 6-Hydroxydopamine
AAPH: 2,20-Azobis(2-amidinopropane) dihydrochloride
ACC: Acetyl-coa carboxylase
ALT: Alanine aminotransferase
AMP: Adenosine 5'-monophosphate
AMPK: AMP-activated protein kinase
AST: Aspartate aminotransferase
ATP: Adenosine triphosphate
C/EBP a: CCAAT/enhancer-binding protein a
CAA: Cellular antioxidant activity assay
CAT: Catalase
C/EBP α: CCAAT/enhancer-binding protein α
CkTcS: Theacrine synthase in *C. Kucha*
CoA: Coenzyme a
CPA: Cyclopentyladenosine
DA: Dopamine
DDPH: Diphenyl-2-picrylhydrazyl
DPCPX: 8-cyclopentyl-1,3-dipropylxanthine
DW: Dry weight
EEG: Electroencephalogram
EMG: Electro-magnetic gun
EMT: Epithelial-to-mesenchymal transition
FABP: Fatty acid-binding protein
FAS: Fatty acid synthase
FAT: Fatty acid translocase
FIA: Freund's incomplete adjuvant
FOXO3: Forkhead Box O3, a protein coding gene, including DNA-binding transcription factor activity and protein kinase binding
FRAP: Ferric reducing ability of plasma
GAPDH: Glyceraldehyde-3-phosphate dehydrogenase
GSH-Px: Glutathione peroxidase
HFD: High fat diet
Hpi: Hour post-infection
IFN: Interferon
IL: Interleukin
LCAD: Lightweight chemical agent detector
LPL: Lipoprotein lipase
LTL: Liver triglyceride lipase
MDA: Malondialdehyde
MPP^+^: 1-methyl-4-phenylpyridinium
MPTP: 1-methyl-4-phenyl-1, 2, 3, 6-tetrahydropyridine
NAFLD: Nonalcoholic fatty liver disease
NF-kB: Nuclear factor-kb
NOAEL: No observed adverse effect level
NREMS: Non-rapid-eye-movement sleep
ORAC: Oxygen radical absorbance capacity
PD: Parkinson's disease
PPAR: Peroxisome proliferator-activated receptor
p-ERK: phosphorylated extracellular regulated protein kinases
p-p38 MARK: phosphorylated mitogen-activated protein kinases
PWS: Preworkout supplements
REMS: Rapid-eye-movement sleep
SAM: S-adenosyl-L-methionine
SCD: Stearoyl-coa desaturase
SIRT3: Sirtuin-3 (SIRT3), a major mitochondria NAD^+^-dependent deacetylase
SOD: Superoxide dismutase
SMAD: SMAD are the proteins required for the transmission of the TGF-β signal to the nucleus.
SREBP-1c: Sterol regulatory element binding transcription factor 1c
TEAC: Trolox equivalent antioxidant capacity
TG: Triglyceride
TMT: Trail making test
TNF: Tumor necrosis factor
TNSALP: Tissue-non-specific alkaline phosphatase
TGF-β: Transforming growth factor-β
TNBC: Triple negative breast cancer.
